# Prevalence and Determinants of Vaccine Hesitancy and Vaccines Recommendation Discrepancies among General Practitioners in French-Speaking Parts of Belgium

**DOI:** 10.3390/vaccines9070771

**Published:** 2021-07-10

**Authors:** Cathy Gobert, Pascal Semaille, Thierry Van der Schueren, Pierre Verger, Nicolas Dauby

**Affiliations:** 1 Department of Infectious Diseases, CHU Saint-Pierre, Université Libre de Bruxelles (ULB), 1000 Bruxelles, Belgium; catgob93@gmail.com; 2 Department of General Medicine, Université Libre de Bruxelles (ULB), 1070 Bruxelles, Belgium; pascal.semaille@stpierre-bru.be; 3 Scientific Society of General Practice, 1060 Bruxelles, Belgium; thierry.vanderschueren@ssmg.be; 4 Southeastern Health Regional Observatory (ORS PACA), 13005 Marseille, France; pierre.verger@inserm.fr; 5 School of Public Health, Université Libre de Bruxelles (ULB), 1070 Bruxelles, Belgium; 6 Institute for Medical Immunology, Université Libre de Bruxelles (ULB), 1070 Bruxelles, Belgium

**Keywords:** vaccine hesitancy, general practice, health-care workers, vaccination

## Abstract

General practitioners (GPs) play a critical role in patient acceptance of vaccination. Vaccine hesitancy (VH) is a growing phenomenon in the general population but also affects GPs. Few data exist on VH among GPs. The objectives of this analysis of a population of GPs in the Belgian Wallonia-Brussels Federation (WBF) were to: (1) determine the prevalence and the features of VH, (2) identify the correlates, and (3) estimate the discrepancy in vaccination’s behaviors between the GPs’ children and the recommendations made to their patients. An online survey was carried out among the population of general practitioners practicing in the WBF between 7 January and 18 March 2020. A hierarchical cluster analysis was carried out based on various dimensions of vaccine hesitancy: perception of the risks and the usefulness of vaccines as well as vaccine recommendations for their patients. A total of 251 GPs answered the survey. The average percentage of moderate to high vaccine hesitancy was 50.6%. Three factors were independently associated with increased risk of vaccine hesitancy: an age <50 years old, having no children, and having no contact with selected vaccine-preventable disease (measles, complicated influenza, chronic hepatitis B (HBV), bacterial meningitis, or cervical cancer) in the past 5 years. VH was associated with controversies on vaccines’ safety. GPs who had vaccinated their children against six diseases (MMR, meningococcus C (MenC), HBV, and HPV) tended not to recommend the same vaccines to their patients. Among GPs with all children vaccinated against HBV, only 37.5% recommended catch-up HBV immunization to their patients. In this small cohort of GP, moderate to high VH was associated with controversies on vaccines’ safety and with specific personal characteristics (age <50, no children, and no recent experience with a serious VPD). As previously reported, GPs have different vaccine prescription attitude toward their patients and children. These findings should be confirmed in larger cohorts.

## 1. Introduction

Vaccination is a simple and effective means of protecting against certain potentially fatal diseases. Vaccination provides both individual and community protection for certain vaccine-preventable diseases and has also broad societal impact that includes increased productivity, positive fiscal impact, and decreased antibiotic consumption [1]. In order to avoid the resurgence of vaccine-preventable diseases (VPD), it is necessary to keep a sufficiently high vaccination coverage.

In Belgium, only the polio vaccine is currently compulsory [2]. Vaccination coverage in the Wallonia-Brussels Federation (WBF), the two French-speaking regions of Belgium, particularly for measles–mumps–rubella (MMR) and for human papillomavirus (HPV) vaccination, is still far from sufficient. For example, vaccine coverage for the second doses of MMR among adolescents was 75% [3]. In the last years, measles outbreaks have been reported both in Brussels and Wallonia regions [4,5].

Vaccine hesitancy (VH) is considered an important cause of under-vaccination [6]. The concept of a matrix of determinants of hesitation, influencing the decision and/or the vaccination methods, has also been proposed in order to group different factors into three impact categories. These categories are contextual, individual, and group, as well as those specific to the vaccine and/or vaccination [7]. In 2016, VH was reported in more than 90% of countries in the world, regardless of socio-economic level [8]. In a 2016 study of 67 countries, Europe had the lowest level of confidence in vaccine safety [9]. Different factors contribute to increased VH: potential side effects, belief that the vaccine could cause the disease it was meant to prevent, doubt about the safety of adjuvants, and overloading the immune system [8].

VH is not limited to patients but also affects healthcare professionals (HCP) [10]. Several causes of VH among HCP have been highlighted: a lack of confidence in the health authorities, the fact of not feeling comfortable transmitting quality information to patients, the fear of serious side effects and doubts regarding the safety of adjuvants and also the efficacy of some vaccines [11].

General practitioners (GPs) play a key role in the promotion and acceptance of vaccination in Belgium as well as in many other countries. Various studies have shown that (1) GPs remain one of the most reliable advisers in the eyes of patients and (2) patients tend to be more reassured when information comes from their doctor [12,13].

In 2014, Verger et al. reported a method to describe and estimate the extent of vaccine hesitancy among GPs. Using a survey that included different dimensions of vaccine hesitancy (self-reported vaccine recommendations, perceptions of vaccine risks and usefulness), they were able to identify three clusters of GP with different grade of VH. GP with the higher degree of VH were less frequently vaccinated and reported more frequent occasional practice of alternative medicine but also less experience with VPD [11].

Interestingly, recent studies have shown a discrepancy in immunization recommendation in a high proportion of GPs toward members of their family, themselves, and their patients [14,15].

There is only a limited amount of data on VH among HCP [13,14,16,17]. In French-speaking parts of Belgium, at present, there is no documented study on this subject.

The objectives of the present study were to (1) estimate VH prevalence in a sample of GPs practicing in the Wallonia-Brussels Federation, (2) identify the factors influencing VH among GPs, and (3) highlight the existence or not of a discrepancy between the vaccination of doctors and their children compared to that of their patients.

## 2. Materials and Methods

### 2.1. Study Design

An online survey aimed at GPs practicing in the Wallonia-Brussels Federation was conducted between 7 January 2020 and 18 March 2020. An online questionnaire was distributed by email to GPs thanks to the collaboration of the Scientific Society of General Practice (SSGP) and the Belgian group of general practitioners (GBO). In addition, several associations of GPs in the Wallonia-Brussels Federation were contacted in order to disseminate the survey to their members by email.

### 2.2. Data Collection

Each GP who agreed to participate in the study answered a questionnaire that included 24 questions (Supplementary materials File S1). This questionnaire was created and standardized by Verger and colleagues [11] For this work, the questionnaire was adapted to match the specificities of the WBF vaccination program (Supplementary materials Table S2). Participants were asked about their personal and professional vaccination practices. They were asked about their views on vaccination and potential barriers to vaccination. The participants gave their opinion on various possible actions pertaining to vaccination. To answer the questions, 4- or 5-point Likert scales were used [18]. The data collection was anonymous. Data were collected using the open-source LimeSurvey software (LimeSurvey GmbH, Hambourg, Germany) hosted on the servers of the Université Libre de Bruxelles (ULB).

### 2.3. Immunization Discrepancies between Family and Patients of General Practitioners

Among GPs with ≥1 child, immunization recommendations of four vaccines (HPV, MMR, meningococcus C (MenC), and Hepatitis B (HBV)) toward their patients were compared with the immunization practice with their family members.

### 2.4. Statistical Analysis

In order to classify population sample according to VH, a previously described method was used [11]. The following characteristics were used to determine VH: belief in the susceptibility of serious adverse effects (six items), doubts about the usefulness of vaccination (two items), and recommendation of vaccination compared to the mean of this population (six items). Likert scores were dichotomized for each of these items. This method allows to assess and grade vaccine-related attitudes and behaviors and is a reflection of the multiples components of VH. A multiple component analysis (MCA) based on the 14 questions was carried out, followed by a hierarchical cluster analysis (HCA), which allowed us to divide the individuals into classes according to their similarities, as previously described [11]. Briefly, MCA is an exploratory technique used to understand the inter-relationships between multiple categorical variables and it allows correlated variables to be combined into continuous factors. These factors are introduced in the HCA, which classifies individuals with similar characteristics into clusters [11]. For the univariate hypothesis tests, Fisher’s exact test was used. For multivariate hypothesis testing, logistic regression was performed. Variables significantly associated with moderate-to-high VH after univariate analysis were used for multivariate analysis. For each predictor of the multivariate analysis, the odds ratios with 95% confidence intervals were calculated. For all analyses, a *p*-value < 0.05 was considered statistically significant. When populations had to be compared, chi-square was used. Statistical analyses were carried out with the XLSTAT software.

## 3. Results

### 3.1. Characteristics of the Study Population

Among the GPs approached during the recruitment period, 251 completed the questionnaire. The profile of the individuals who refused to participate in the study is unknown. The characteristics of the GPs included are listed in Table 1. Most participants were female. The majority of the participants had participated in an immunization course program in 2019 and had an experience with the selected VPD in the past 5 years. Alternative medicine practice was reported by 18.3% of the participants. As compared to the GPs reported in the WBF in 2018, there was a higher proportion of women and GPs < 50 years old represented (Supplementary Table S1). The place of practice was also different, indicating that the population in this study was not representative of the GP population of the WBF.

### 3.2. Definition and Prevalence of Vaccine Hesitancy

In this sample of the population of GPs, two groups were identified based on the perceived association between vaccines and side effects, perceived usefulness of vaccines, and frequency of recommendation (Table 2).

The first group included physicians who were least hesitant about vaccination (*n* = 124), the second consisted of “moderately to highly hesitant” physicians (*n* = 127). In this group, 111 were moderately hesitant and 16 were highly hesitant. For ease of analysis, these two classes have been grouped together via hierarchical cluster analysis.

The more hesitant group had more doubts about the usefulness of vaccines recommend by the authorities (*p* = 0.07). The moderate to highly hesitant group significantly recommended less frequently the six vaccines listed in the survey than the less hesitant group. The most important difference in terms of recommendation was for the hepatitis B virus (HBV) vaccine. Indeed, only 21.3% of physicians with moderate to high VH recommended this vaccination compared to 92.7% of physicians with little or no VH.

The more hesitant group more frequently assumed a link between the vaccines and the potential side effects they might cause compared to the less hesitant groups. This difference was significant for three situations: a link between the HBV and multiple sclerosis, a link between the HPV vaccine and multiple sclerosis, and a link between adjuvants present in vaccines and long-term complications. The most frequent association made by this population was the presence of adjuvants in vaccines as possibly causing long-term complications. This perception was present in 17/251 (6.8%) individuals in this sample. This association was much greater in the most hesitant group (11.8%) than in the least hesitant group (1.6%) (*p* = 0.002). There was no significant difference for the other three situations (Table 2).

### 3.3. Determinants of Moderate to High Vaccine Hesitancy

The analysis of the determinants for moderate to high VH is given in Table 3.

Among the variables studied, four were significantly associated with moderate to high VH in the univariate analysis: age, having or not having children, whether or not they have participated in immunization training in the past 5 years, and whether or not they have had experience with at least one of the five selected vaccine-preventable diseases (measles, complicated influenza, chronic HBV, bacterial meningitis or cervical cancer) Multivariate analysis identified three determinants of moderate to high VH among GPs: younger age, not having experience with a selection of vaccine-preventable disease recently, as well as having no children. Having not followed an immunization course in the last 5 years was borderline significant (*p* = 0.051). No influence of personal vaccination history on VH was found.

### 3.4. Discrepancies between General Practitioners Family Immunization Practice and Patient’s Immunization Recommendations

The majority (59.3%) of GPs in this population sample had at least one child between the ages of 2 and 25. Among these physicians, 131/149 (87.9%) exhibited at least one discordant behavior. Most GPs with discordant behavior tended to vaccinate their children even though they did not always recommend the vaccine to their patients (Table 4).

GPs who had no or only some of their children vaccinated tended not to recommend some vaccines to their patients (MenC, HPV, and HBV) (Table 4). Overall, a vaccine discordance was found for the four vaccines studied (HPV, MMR, MenC, and HBV). This difference in vaccination behavior between GPs patients and vaccination of their children was highly significant for all the vaccines studied. The discordance was highest for the HBV vaccine. Overall, 36.2% of physicians still recommended catch-up vaccination in the event of non-vaccination in infancy, while 96.6% of physicians had vaccinated all their children against this pathology. Among practitioners who had vaccinated all their children against hepatitis B, 62.5% did not systematically recommend a catch-up vaccination for this vaccine. The smallest discordance related to the HPV vaccine was found in teenage girls. For this vaccine, 23.4% of doctors who vaccinated their daughters did not systematically recommend vaccination for their patients.

## 4. Discussion

### 4.1. Prevalence and Degree of Vaccine Hesitancy

In this study, we characterized and assessed the prevalence of VH in a population of GPs practicing in WBF, the French-speaking part of Belgium using a method previously used in a survey among French GPs. This method uses cluster analysis of different dimensions of VH to assess the extent but also the correlates of VH [11]. In the present work, two categories of physicians according to their degree of VH (none to low and moderate to high) were identified as well as different determinants associated with moderate to high VH.

The prevalence of moderate to high hesitation was 50.6%. In comparison, Verger and colleagues found, in a population of French GPs, moderate to high VH in 14% of their population [11]. Although we used the same items and analysis method to assess the extent of VH, this high prevalence of moderate-to-high VH should be taken with caution. Indeed, the answers given by GPs with moderate to high VH in our study to the different questions related to practices and opinions about vaccination were more nuanced as compared to the study by Verger et al. In our study, the most hesitant population was less sensitive to controversies, had less doubt about vaccine usefulness, and more frequently recommended vaccination, as compared to the study performed among French GPs [11]. This limitation is inherent to the methods used. Indeed, the method allows the identification of clusters within a specific population sample. The identification of the clusters is based on a gradation of VH. Gradation of VH is much less contrasted in our sample as compared to the French GP sample, with under representation of highly reluctant GP (Table 5).

The Belgian GPs included in this survey therefore seemed to exhibit different vaccination behaviors from French GPs and were generally less hesitant than in France. This is in line with the results of a large study carried out in 67 countries revealed regional differences in terms of VH among the general population [9]. In a recent survey performed in October–November 2020 regarding acceptance of COVID-19 vaccine, we also observed differences between French and Belgian GPs, and nurses from Canada [17].

Among the category of least hesitant GPs, 96.8% said they were very favorable to the vaccination as compared to 87.4% among the most hesitant GPs. The remaining individuals in both groups were rather favorable to the vaccination. There were no general practitioner opposed to vaccination in general. Despite these rather encouraging results, it is surprising to note that 8.4% of the participants believed that certain vaccines recommended by the authorities were not useful. The proportion of GPs judging certain recommended vaccines to be unnecessary was higher in the moderately to highly hesitant group but not statistically significant (11.8% vs. 4.8%, *p* = 0.07) probably because of the limited size of our sample. This underlines the importance of active communication strategies by authorities about current and future immunization strategies. Previous studies have shown that increased awareness and knowledge about vaccination were associated with increased willingness of HCP to recommend vaccination [16].

A significant number of GPs included in the study showed demonstrated worries about the possible association of vaccination and adverse health impact. The association between adjuvants and the long-term health complications was the most common, although there is no scientific evidence, to date, to support such claim [19]. Of note, both groups of GPs surveyed associated the influenza vaccine with the risk of Guillain-Barré as well as Pandemrix with an increased risk of narcolepsy. These two associations have, unlike the other situations studied, been reported [20,21]. Some HCP may feel uncertain about the safety and efficacy of certain vaccines, including recently with the COVID-19 vaccines [17]. Such uncertainties were associated with a decrease in the frequency of recommending vaccination to patients [22,23] Our findings highlight the importance of appropriate training about vaccine side effects during medical education. Accordingly, a study performed among French medical students found that a significant proportion of medical students does not feel prepared to communicate about side effects of vaccines [24].

For all the vaccine situations studied, the group with moderate to high VH recommended vaccination less often than the group with no or low hesitation. In the population studied, it is important to note that the recommendation of the vaccine against meningococcus C was particularly low, whether during the primary vaccination or during catch-up vaccination. This is of concern given the critical role of group immunity which has led to a decrease in the incidence of invasive meningococcal C infections [25]. In the context of catch-up vaccination, this low recommendation frequency could also result from the lack of reimbursement of this vaccine in Belgium despite guidelines by the Superior Scientific Health Council [2].

### 4.2. Determinants of Moderate to High Vaccine Hesitancy

Three risk factors for vaccine hesitancy were identified in our survey of Belgian GPs: being under 50 years old, not having children, and no reported experience with a selection VPD in the past five years. Contrary to previously published studies [26], a younger age was associated with a higher level of VH. This will need to be studied in further studies on a larger scale in order to assess more precisely the impact of age on VH. As mentioned above, recent research among medical students has highlighted a significant rate of unpreparedness regarding knowledge about vaccine-related issues [24]. Less hesitancy was observed with doctors with progeny.

Lack of experience with a selection of VPD in general medicine also increased VH. This result is consistent with that of Verger et al. [11]. A doctor’s own experience, the fact that he may have lived through a sometimes-dramatic situation can influence the opinion he may have on vaccination. In this study, individuals who had professional experience with meningitis in the past five years often or always recommended the primary meningococcal C vaccination in 77.1% of cases, versus only 68.5% of those without such experience (*p*-value < 0.001).

This study showed that there is a tendency for VH to increase among GPs who have not participated in vaccination training in the past five years. The association was borderline significant. A study including more participants should be carried out in order to more precisely assess the role of training on VH. Previous research has identified lack of training as a factor associated with a decrease of the frequency of healthcare professional’s vaccination recommendations to their patients [16]. As official vaccine recommendations vary regularly, it seems important that physicians are regularly updated with new information. However, the need for a training on vaccination was not different between GPs with none-to-low and moderate-to-high VH.

### 4.3. Vaccine Discordance of General Practitioners between the Vaccination of Their Children and Their Professional Recommendations

The discrepancy analysis showed the existence of at least one discordant behavior in 87.9% of GPs with at least one child aged between 2 and 25 years. Two other studies carried out on vaccine divergence showed discordant behavior in 45.7 to 60% of the individuals questioned [14,15]. As stated above, having a child is a factor associated with reduced GPs’ vaccine hesitancy. In our study we also found that doctors tended to immunize their own children much more frequently than they recommended for their patients, for all four vaccines studied. This is of concern for hepatitis B vaccination. A French study showed that a decline in vaccination among adolescents was associated with an increase in the incidence of HBV in adults [27]. The lack of recommendation of GPs could be related in part to the phenomenon of VH in patients. Healthcare professionals represent the most trusted source of information about immunization for the population [16,28]. While communication has been shown to be an essential tool in patient acceptance of vaccination [16,29], physicians may feel helpless in the face of the situation and fail to respond adequately to their patient’s questions [30]. Other reasons could play an important role in the existing vaccine discordance like unpreparedness and time constraints [16].

There were several limitations in this work. First, participants answered the questionnaire based on self-reported behaviors. This can lead to reporting and/or social desirability bias even though the questionnaire is anonymous. In addition, a sample selection bias most likely existed because of the voluntary nature of the participation. Thus, the sample might not be an adequate representation of the whole population of GPs in the French speaking parts of Belgium. In fact, some characteristics of the sample studied were different from the population of GPs in the WBF (Supplementary Table S1). The sample size is small and larger study should be performed to confirm the findings. We did not ask which vaccines recommended by the authorities some GP found not useful. More studies are needed to identify if specific vaccines are considered as such and the reasons associated with such feeling.

The possibility also exists that those who are interested and feeling concerned and/or worried by the current problem of VH would have been more willing to answer this questionnaire. However, this is the first study on this issue in Belgium. It could therefore serve as a starting point for a larger study in Belgium and in which the practices and opinions of doctors in various countries of the European Union could be compared.

## 5. Conclusions

In the present study, three independent risk factors increasing VH among physicians have been identified: age under 50, not having children, and not having recent experience with a selection of VPD. The group with increased VH were more susceptible to controversies linked to vaccines such as long term impact of adjuvants or a relationship between HBV vaccine and multiple sclerosis. The GPs with high VH recommended less frequently different vaccines to at-risk populations. This at-risk group should receive priority support and targeted training to help control VH. Training should also be improved for all general practitioners, whether during their basic training but also during continuing education. The emphasis should be on both evidence-based training of current immunization recommendations, existing vaccine controversies, and how to defeat them. The significant discrepancy between the vaccination of doctors’ children and that of their patients suggests that GPs, whether hesitant or not, are possibly not sufficiently armed to deal with the growing vaccine hesitancy of patients.

## Figures and Tables

**Table 1:** Characteristics of the sample of Belgian GPs who answered the surveys between January to March 2020 (*n* = 251).

Characteristics	Numbers (%)
Personal Characteristics	
Sex	
Men	103/251 (41.0%)
Women	148/251 (59.0%)
Age	
25–34	85/251 (33.8%)
35–49	52/251 (20.7%)
50–64	82/251 (32.7%)
65 and above	32/251 (12.8%)
Office location (2 missing values)	
Brussels-Regio	56/249 (22.5%)
Walloon Brabant	52/249 (20.9%)
Hainaut	79/249 (31.7%)
Namur Province	24/249 (9.6%)
Liège Province	30/249 (12%)
Luxembourg Province	8/249 (3.2%)
Professional Characteristics	
Type of practice	
Solo	104/251 (41.4%)
Association	104/251 (41.4%)
Medical Center	47/251 (18.3%)
Alternative medicine offered	
Yes	46/251 (18.3%)
No	205/251 (81.7%)
Participation in immunization training course in 2019	
Yes	194/251 (77.3%)
No	57/251 (22.7%)
Feels a need for training on vaccination	
Yes	133/251 (53%)
No	118/251 (47%)
Vaccination Experience	
Experience with a selected vaccine-preventable disease in the past 5 years (measles, complicated influenza, chronic hepatitis B (HBV), bacterial meningitis, or cervical cancer)	
Yes	222/251 (88.4%)
No	29/251 (11.6%)
Had a patient with severe disease potentially related to vaccination	
Yes	27/251 (10.8%)
No	224/251 (89.2%)
Opinion about Vaccination	
Favorable to vaccination in general	
Very Favorable	231/251 (92%)
Rather Favorable	20/251 (8%)
Rather Not Favorable	0/251 (0%)
Not Favorable	0/251 (0%)
Think patients must be convinced to get vaccinated even if they are hesitant	
Yes	115/251 (45.8%)
Rather Yes	129/251 (51.4%)
Rather Not	7/251 (2.8%)
No	0/251 (0.0%)
Personal Vaccination History	
Influenza vaccination, season 2018–2019	
Yes	198/251 (78.9%)
No	53/251 (21.1%)
Last diphtheria–tetanus–pertussis dose/booster	
<10 years	218/251 (86.8%)
10–20 years	26/251 (10.4%)
>20 years	6/251 (2.34%)
Unknown	1/251 (0.4%)
Hepatitis B vaccination	
Yes, 3 or more doses	226/251 (90%)
Yes, less than 3 doses	10/251 (4%)
Never or do not remember	10/251 (4%)
Not concerned	5/251 (2%)

**Table 2:** Characteristics of generalist physicians based on their practices and their opinions on vaccination. Vaccine hesitancy degree was determined after hierarchical cluster analysis.

	Vaccine Hesitancy			
	None to Low *N* = 124	Moderate to High *N* = 127	All *N* = 251	*p*-Value (Fisher)
Perceived link between vaccines and potential severe side-effects (somewhat likely, very likely)				
Influenza and Guillain-Barré	18/124 (14.5%)	26/127 (20.5%)	17.5%	0.25
Hepatitis B and multiple sclerosis	1/124 (0.8%)	8/127 (6.3%)	3.6%	0.04
Human papillomavirus (HPV) and multiple sclerosis	0/124 (0.0%)	6/127 (4.7%)	2.4%	0.03
Aluminum and Alzheimer’s disease	3/124 (2.4%)	5/127 (3.9%)	3.2%	0.72
Pandemrix and narcolepsy	13/124 (10.5%)	5/127 (3.9%)	7.2%	0.052
Adjuvants and long-term complications	2/124 (1.6%)	15/127 (11.8%)	6.8%	0.002
Perceived usefulness of vaccines (mostly agree, totally agree)				
Some vaccines recommended by authorities are not helpful	6/124 (4.8%)	15/127 (11.8%)	8.4%	0.07
Children are vaccinated against too many diseases	6/124 (4.8%)	7/127 (5.5%)	5.2%	0.87
Referral frequency (often, always)				
Measles–mumps–rubella (MMR) in unimmunized adolescents and young adults	120/124 (96.8%)	92/127 (72.4%)	84.5%	<0.0001
Meningococcus C at 13–15 months	110/124 (88.7%)	65/127 (51.2%)	69.7%	<0.0001
Catch-up for meningococcus C	80/124 (64.5%)	2/127 (1.6%)	32.7%	<0.0001
Human Papillomavirus (HPV) in girls 11–14 years old	123/124 (99.2%)	115/127 (90.6%)	94.8%	<0.01
Catch-up hepatitis B in adolescents	115/124 (92.7%)	27/127 (21.3%)	56.6%	<0.0001
Flu in people with diabetes <65 years	123/124 (99.2%)	106/127 (83.5%)	91.2%	<0.001

**Table 3:** Factors associated with moderate to high vaccine hesitancy (VH) in a population of general practitioners (*n* = 251).

Characteristics (*n* = 251)	VH None to Low *n* (%)	VH Moderate to High *n* (%)	OR (CI 95%)	*p*-Value	AOR (CI 95%)	*p*-Value
Personal Characteristics						
Sex						
Men	52/124 (41.94%)	51/127 (40.2%)	1.08	0.80		
Women	72/124 (58.06%)	76/127 (59.8%)	(0.63–1.84)			
Age						
≥50 years’ old	68/124 (54.84%)	46/127 (36.2%)	2.14		1.76	
<50 years’ old	56/124 (45.16%)	81/127 (63.8%)	(1.25–3.66)	<0.01	(1.02–3.03)	0.04
At least one child						
Yes	92/124 (74.19%)	57/127 (46%)	3.53		3.11	
No	32/124 (25.81%)	70/127 (56%)	(2.00–6.25)	<0.001	(1.79–5.40)	<0.001
*Professional Characteristics*						
Offer alternative medicine						
Yes	22/124 (17.74%)	24/127 (18.9%)	0.93			
No	102/124 (82.26%)	103/127 (81.1%)	(0.46–1.85)	0.87		
Immunization training course <5 years						
Yes	104/124 (83.87%)	90/127 (70.9%)	2.14		1.92	
No	20/124 (16.13%)	37/127 (29.1%)	(1.11–4.17)	0.02	(1.00–3.70)	0.051
*Vaccination Experience*						
Experience with a selected vaccine-preventable disease (measles, complicated influenza, chronic hepatitis B (HBV), bacterial meningitis or cervical cancer)						
Yes	118/124 (95.16%)	104/127 (81.9%)	4.35		3.23	
No	6/124 (4.84%)	23/127 (18.1%)	(1.63–13.50)	0.001	(1.24–8.42)	0.02
Experience with a serious health problem potentially related to the vaccine						
Yes	9/124 (7.26%)	18/127 (14.2%)	0.47			
No	115/124 (92.74%)	109/127 (85.8%)	(0.18–1.17)	0.1		
*Personal Vaccination History*						
Influenza vaccination season 2018–2019						
Yes	98/124 (79.03%)	100/127 (78.7%)	1.02			
No	26/124 (20.97%)	27/127 (21.3%)	(0.53–1.96)	1		
Last DTP dose						
<10 years	109/124 (87.90%)	109/127 (85.8%)	1.20			
≥10 years or does not remember	15/124 (12.10%)	18/127 (14.2%)	(0.54–2.70)	0.71		
Hepatitis B vaccination						
Yes, 3 doses or more	111/124 (89.52%)	115/127 (90.6%)				
Less than 3 or does not remember	10/124 (8.06%)	10/127 (7.9%)				
Not concerned	3/124 (2.42%)	2/127 (1.5%)		0.94		

**Table 4:** Recommendations by general practitioners for children and their patients.

	Decision for Their Children		
Frequency of Recommendation to Their Patients	All Are Vaccinated	None or Some Are Vaccinated	Total
Measles–mumps–rubella (for adolescents and young adults)			
Always	101/147 (68.7%)	2/2 (100%)	103/149 (69.1%)
Not always	46/147 (31.3%)	0/2 (0%)	46/149 (30.9%)
Meningococcus C at 15 months *			
Always	84/139 (60,43%)	1/8 (12.5%)	85/147 (57.8%)
Not always	55/139 (39,57%)	7/8 (87.5%)	62/147 (42.2%)
Human papillomavirus (HPV) in girls 11–14 years old **			
Always	59/77 (76.6%)	0/6 (0%)	59/83 (71.1%)
Not always	18/77 (23.4%)	6/6 (100%)	24/83 (28.9%)
Catch-up hepatitis B vaccine			
Always	54/144 (37.5%)	0/5 (0%)	54/149 (36.2%)
Not always	90/144 (62.5%)	5/5 (100%)	95/149 (63.8%)

* Missing 2 data points, ** Only 83 doctors.

**Table 5:** Comparison of the typology of general practitioners with moderate to high vaccine hesitancy identified in the present study and the original study by Verger et al.

	Vaccine Hesitancy (%)		
	Belgian GP (Present Study)	French GP (from the Study by Verger et al. [11])	
	Moderate to High *N* = 127	Moderate *N* = 166	High *N* = 56
Perceived link between vaccines and potential severe side-effects (somewhat likely, very likely)			
Influenza and Guillain-Barré	20.5	29.9	66.2
Hepatitis B and multiple sclerosis	6.3	30.3	82.8
Human papillomavirus (HPV) and multiple sclerosis	4.7	15.2	70.9
Aluminum and Alzheimer’s disease	3.9	28.8	46.4
Pandemrix and narcolepsy	3.9	27.4	50.5
Adjuvants and long-term complications	11.8	48.2	88.5
Perceived usefulness of vaccines (mostly agree, totally agree)			
Some vaccines recommended by authorities are not helpful	11.8	40.1	60.4
Children are vaccinated against too many diseases	5.5	36.5	62.4
Referral frequency (often, always)			
Measles–mumps–rubella (MMR) in unimmunized adolescents and young adults	72.4	55.8	52.6
Meningococcus C at 15 months	51.2	52.8	30.6
Catch-up for meningococcus C	1.6	36.2	20.8
Human papillomavirus (HPV) in girls 11–14 years old	90.6	46.9	24.5
Catch-up hepatitis B in adolescents	21.3	41.5	29.7
Flu in people with diabetes <65 years	83.5	69.9	47.5

## Data Availability

The data presented in this study are available on request from the corresponding author.

## Supplementary Materials

The following are available online at https://www.mdpi.com/article/10.3390/vaccines9070771/s1, File S1: Survey, Table S1: Comparison of the population of general practitioners practicing in the Wallonia-Brussels Federation (approved generalist physicians + generalist physicians in training) with the sample represented in the study. Table S2: Vaccination calendar in French-Speaking Belgium (Brussels & Wallonia).

## References

[1] Bloom D.E., Fan V.Y., Sevilla J.P. (2018). The broad socioeconomic benefits of vaccination. Sci. Transl. Med..

[2] Conseil Supérieur de la Santé (CSS) (2019). CSS 9141—Calendrier Vaccinal de Base Recommandé Par Le CSS.

[3] Wyndham-Thomas C., Grammens T., Litzroth A., Sizaire V., Boon N., Muyldermans G., Lernout T. Maladies infectieuses à prévention vaccinale. Synthèse annuelle 2017. Bruxelles, Belgique: Sciensano; 2019. Numéro de rapport: D/2019/14.440/22. https://www.sciensano.be/sites/default/files/maladies_infectieuses_pediatriques_a_prevention_vaccinale._synthese_annuelle_2017.pdf.

[4] Grammens T., Schirvel C., Leenen S., Shodu N., Hutse V., Da Costa E.M., Sabbe M. (2017). Ongoing measles outbreak in Wallonia, Belgium, December 2016 to March 2017: Characteristics and challenges. Eurosurveillance.

[5] Cornelissen L., Grammens T., Leenen S., Schirvel C., Hutse V., Demeester R., Swennen B., Asikainen T., Wyndham-Thomas C. (2020). High number of hospitalisations and non-classical presentations: Lessons learned from a measles outbreak in 2017, Belgium. Epidemiol. Infect..

[6] Gostin L., Hodge J.G., Bloom B.R., El-Mohandes A., Fielding J., Hotez P., Kurth A., Larson H.J., Orenstein W., Rabin K. (2020). The public health crisis of underimmunisation: A global plan of action. Lancet Infect. Dis..

[7] MacDonald N.E. (2015). Vaccine hesitancy: Definition, scope and determinants. Vaccine.

[8] Lane S., MacDonald N.E., Marti M., Dumolard L. (2018). Vaccine hesitancy around the globe: Analysis of three years of WHO/UNICEF Joint Reporting Form data-2015–2017. Vaccine.

[9] Larson H.J., De Figueiredo A., Xiahong Z., Schulz W.S., Verger P., Johnston I., Cook A.R., Jones N.S. (2016). The State of Vaccine Confidence 2016: Global Insights Through a 67-Country Survey. EBioMedicine.

[10] Karafillakis E., Larson H.J. (2018). The paradox of vaccine hesitancy among healthcare professionals. Clin. Microbiol. Infect..

[11] Verger P., Collange F., Fressard L., Bocquier A., Gautier A., Pulcini C., Raude J., Peretti-Watel P. (2016). Prevalence and correlates of vaccine hesitancy among general practitioners: A cross-sectional telephone survey in France, April to July 2014. Eurosurveillance.

[12] Karafillakis E., Dinca I., Apfel F., Cecconi S., Wűrz A., Takacs J., Suk J., Celentano L.P., Kramarz P., Larson H.J. (2016). Vaccine hesitancy among healthcare workers in Europe: A qualitative study. Vaccine.

[13] Harrison N., Poeppl W., Miksch M., Machold K., Kiener H., Aletaha D., Smolen J.S., Forstner C., Burgmann H., Lagler H. (2018). Predictors for influenza vaccine acceptance among patients with inflammatory rheumatic diseases. Vaccine.

[14] Killian M., Detoc M., Berthelot P., Charles R., Gagneux-Brunon A., Lucht F., Pulcini C., Barbois S., Botelho-Nevers E. (2016). Vaccine hesitancy among general practitioners: Evaluation and comparison of their immunisation practice for themselves, their patients and their children. Eur. J. Clin. Microbiol. Infect. Dis..

[15] Agrinier N., Le Maréchal M., Fressard L., Verger P., Pulcini C. (2017). Discrepancies between general practitioners’ vaccination recommendations for their patients and practices for their children. Clin. Microbiol. Infect..

[16] Paterson P., Meurice F., Stanberry L.R., Glismann S., Rosenthal S.L., Larson H.J. (2016). Vaccine hesitancy and healthcare providers. Vaccine.

[17] Verger P., Scronias D., Dauby N., Adedzi K.A., Gobert C., Bergeat M., Gagneur A., Dubé E. (2021). Attitudes of healthcare workers towards COVID-19 vaccination: A survey in France and French-speaking parts of Belgium and Canada, 2020. Eurosurveillance.

[18] Likert R. (1932). A Technique for the Measurement of Attitudes. Arch. Psychol..

[19] Plotkin S.A., Offit P.A., DeStefano F., Larson H.J., Arora N.K., Zuber P.L., Fombonne E., Sejvar J., Lambert P.H., Hviid A. (2020). The science of vaccine safety: Summary of meeting at Wellcome Trust. Vaccine.

[20] Sarkanen T.O., Alakuijala A.P., Dauvilliers Y., Partinen M. (2018). Incidence of narcolepsy after H1N1 influenza and vaccinations: Systematic review and meta-analysis. Sleep Med. Rev..

[21] Arias L.M., Sanz R., Sainz-Gil M., Treceño C., Carvajal A. (2015). Guillain-Barré syndrome and influenza vaccines: A meta-analysis. Vaccine.

[22] Salmon D.A., Dudley M.Z., Glanz J.M., Omer S.B. (2015). Vaccine Hesitancy. Am. J. Prev. Med..

[23] Karlsson L.C., Lewandowsky S., Antfolk J., Salo P., Lindfelt M., Oksanen T., Kivimäki M., Soveri A. (2019). The association between vaccination confidence, vaccination behavior, and willingness to recommend vaccines among Finnish healthcare workers. PLoS ONE.

[24] Kernéis S., Jacquet C., Bannay A., May T., Launay O., Verger P., Pulcini C., Abgueguen P., Ansart S., Bani-Sadr F. (2017). Vaccine Education of Medical Students: A Nationwide Cross-sectional Survey. Am. J. Prev. Med..

[25] Conseil Supérieur de la Santé (CSS) (2019). Vaccination Contre Le Méningocoque—Avis N°9485.

[26] Le Marechal M., Fressard L., Agrinier N., Verger P., Pulcini C. (2018). General practitioners’ perceptions of vaccination controversies: A French nationwide cross-sectional study. Clin. Microbiol. Infect..

[27] Ramière C., Roche L., Scholtès C., Iwaz J., Saison J., Ecochard R., André P. (2016). Evolution of the incidence of hepatitis B virus infection and immunization rates in a large French cohort born between 1960 and 1994. Clin. Microbiol. Infect..

[28] Smith P.J., Kennedy A.M., Wooten K., Gust D.A., Pickering L.K. (2006). Association Between Health Care Providers’ Influence on Parents Who Have Concerns About Vaccine Safety and Vaccination Coverage. Pediatrics.

[29] Larson H.J., Cooper L.Z., Eskola J., Katz S.L., Ratzan S. (2011). Addressing the vaccine confidence gap. Lancet.

[30] McRee A.-L., Gilkey M.B., Dempsey A.F. (2014). HPV Vaccine Hesitancy: Findings from a Statewide Survey of Health Care Providers. J. Pediatr. Health Care.

