# Supplementary material for: Prevalence and Determinants of Vaccine Hesitancy and Vaccines Recommendation Discrepancies among General Practitioners in French-Speaking Parts of Belgium
Source: Vaccines (Basel). 2021 Jul 10;9(7):771. doi: 10.3390/vaccines9070771 (PMC8310255; doi:10.3390/vaccines9070771)
Supplement: Supplementary file 1 [file vaccines-09-00771-s001.zip › File S1-survey.pdf]

File S1

Part 1 : Introduction

A1. How old are you ?

- 25-34
- 35-49
- 50-64
- 65 and over

A2. Gender?

- Woman
- Male

A3. In which province do you practice?

- Brussels
- Walloon Brabant
- Hainaut
- Province of Namur
- Province of Liege
- Province of Luxembourg

A4. What is your practice style?

- Solo
- Association of doctors
- Medical center

A5. Do you occasionally practice alternative medicine (for example : acupuncture, homeopathy)?

- Yes
- No

A6. With regard to vaccination in general in your daily practice, are you:

- Not at all favorable
- Rather not favorable
- Rather favorable
- Very favorable

A7. On the subject of infectious diseases and/or vaccination, how many half-days or evenings of training have you attended in the last 12 months?

/_ _ /

A8. Do you feel a need for training on vaccination?

- Yes
- No
-

Part B: Personal and family vaccination practices

B1. Have you personally had the seasonal influenza vaccine for the season of 2018-2019:

- Yes
- No
- Can't remember

B2. When was your last diphtheria-tetanus-pertussis booster?

- Under 10 years old
- 10-20 years
- More than 20 years
- Does not remember

B3. Have you been vaccinated against hepatitis B?

- Yes, 3 or more doses
- Yes, less than 3 doses
- No, doesn't remember
- Not relevant (history of hepatitis B)

B4. If 1 child aged 2 to under 25 (inclusion questionnaire):

<u>You have a child between 2 and 25 years old: have you had him/her vaccinated against:</u>

- Hepatitis B (yes / no)
- Meningococcus C (yes / no)
- MMR (yes / no)

B5. If more than 1 child aged 2 to under 25 (inclusion questionnaire):

<u>You have children between 2 and 25 years old: have you had them vaccinated against:</u>

- Hepatitis B (yes all / yes some / none)
- Meningococcus C (yes all / yes some / none)
- MMR (yes all / yes some / none)

B6. If 1 girl aged 11 to 25 (inclusion questionnaire)

<u>You have a daughter between 11 and 25 years old: have you had her vaccinated against HPV?</u>

- Yes
- No but you're going to do it
- No, because it did not fit into the indications
- No, you do not intend to do it for another reason

B7. If> 1 girl aged 11 to 25 (inclusion questionnaire)

<u>You have daughters between the ages of 11 and 25: have you had them vaccinated against HPV:</u>

- Yes all
- Yes some
- No, but you are going to do it
- No, because they did not fit into the indications
- No, you do not intend to do it for other reasons

<u>Part C: Vaccination practices in general medicine</u>

C1. Do you propose the following vaccines:

- MMR for non-immune adolescents or young adults: never, sometimes, often, always
- Meningococcus C catch-up from 2 to 24 years old
- Meningococcus C in infants aged 12 months
- HPV in girls aged 11-14
- Hepatitis B vaccine as a catch-up for adolescents

- Seasonal flu vaccine for adults with diabetes under 65 years of age

C2. In your experience, during the last 5 years, have you been confronted with the following situations:

- Measles in adolescents or young adults: yes / no
- Chronic hepatitis B of recent discovery
- Hospitalization for complications of a seasonal influenza
- Bacterial meningitis
- Cervical cancer

Part D: Your views on vaccination and some vaccines

D1. Do you feel that your role in relation to the vaccination of your patients is to encourage them to be vaccinated even when they are reluctant?

- No
- Rather not
- Rather yes
- Yes

D2. Do you feel that you can easily get your patients to adhere to vaccination in the following situations? (To be completed according to your practice, it is not mandatory to answer all the sub-questions)

- Against seasonal influenza in people with diabetes under 65: no, rather no, rather yes, yes
- Against meningococcal C meningitis in adolescents and young adults
- Against papillomavirus in girls aged 11-14 years
- Against hepatitis B in unvaccinated adolescents
- Against measles-mumps and rubella in adolescents or young adults

D3. In your opinion, are the following vaccines likely to cause the following diseases?

- Seasonal influenza vaccine and Guillain-Barré syndrome: not at all likely, somewhat unlikely, somewhat likely, very likely, doesn't know
- Vaccine against hepatitis B and multiple sclerosis
- Vaccines containing aluminium and Alzheimer's disease
- Pandemrix vaccine against pandemic influenza H1N1 (2009) and narcolepsy
- Vaccine against papillomavirus and multiple sclerosis

D4. Do you yourself think that the presence of adjuvants in vaccines is associated with long-term complications:

- No
- Rather not
- Rather yes
- Yes

D5. Have you been confronted at least once with a SERIOUS health problem (e.g resulting in hospitalization, incapacity, etc.) potentially linked to vaccination in one of your patients?

- Yes
- No

D6. If yes to D5: Did you notify the health authorities?

- Yes
- No

D7. Do you agree with the following propositions?

- Today, some vaccines recommended by the authorities are unnecessary
- Children are vaccinated against too many diseases
