# Supplementary material for: Prevalence and Determinants of Vaccine Hesitancy and Vaccines Recommendation Discrepancies among General Practitioners in French-Speaking Parts of Belgium
Source: Vaccines (Basel). 2021 Jul 10;9(7):771. doi: 10.3390/vaccines9070771 (PMC8310255; doi:10.3390/vaccines9070771)
Supplement: Supplementary file 1 [file vaccines-09-00771-s001.zip › Table S1.pdf]

Table S1. Comparison of the population of General practitioners practicing in the Wallonia-Brussels Federation (approved generalist physicians + generalist physicians in training) with the sample represented in the study.

	GP's sample studied N=251	Total GP Population WBF (2018) N=8111	p-value (X^2)
**Age**			
- <50 years old	137	3343	<0.001
- ≥ 50 years old	114	4768	
**Sex**			
- Men	103	4456	<0.001
- Women	148	3655	
**Office Location***			
- Brussels	56	1873	<0.001
- Walloon Brabant	52	902	
- Hainaut	79	1956	
- Liège	30	1914	
- Namur	24	1005	
- Luxembourg	8	461	

***1 data point missing for the population studied**

Source : Mahieu T *et al*. STATISTIQUES ANNUELLES DES PROFESSIONNELS DES SOINS DE SANTÉ EN BELGIQUE Nombre de Professionnels en Droit d'Exercer au 31/12/2018 et Influx 2018. https://organesdeconcertation.sante.belgique.be/. Available at: <https://organesdeconcertation.sante.belgique.be/fr/documents/hwf-statan-2018> .
