# Supplementary material for: Prevalence and Determinants of Vaccine Hesitancy and Vaccines Recommendation Discrepancies among General Practitioners in French-Speaking Parts of Belgium
Source: Vaccines (Basel). 2021 Jul 10;9(7):771. doi: 10.3390/vaccines9070771 (PMC8310255; doi:10.3390/vaccines9070771)
Supplement: Supplementary file 1 [file vaccines-09-00771-s001.zip › Table S2.pdf]

Table S2: Vaccination calendar in French-Speaking Belgium (Brussels & Wallonia)

	2 months	3 months	4 months	12 months	13-15 months	5-6 years	7-8 years	13-14 years	15-16 years	Pregnant women	Every 10 years	≥65 years
**Poliomyelitis**	x	x	x		x	x						
**Diphtheria**	x	x	x		x	x			x	x	x	
**Tetanus**	x	x	x		x	x			x	x	x	
**Whooping cough**	x	x	x		x	x			x	x	x	
**Haemophilus influenza type b**	x	x	x		x							
**Hepatitis B**	x	x	x		x							
**Measles, Rubella, Mumps**				x			x					
**Menincococcus C**					x							
**Pneumococcus**	PCV13		PCV13	PCV13								Pneumo23
**Rotavirus**	x	x	(x)									
**Human Papillomavirus**									x			
**Influenza**										x		x

Source: https://www.health.belgium.be/sites/default/files/uploads/fields/fpshealth_theme_file/20210603_fiche_9606_calendrier_vaccinal_css_2021_0.pdf
